# Constructal approach to bio-engineering: the ocular anterior chamber temperature

**DOI:** 10.1038/srep31099

**Published:** 2016-08-05

**Authors:** Umberto Lucia, Giulia Grisolia, Daniela Dolcino, Maria Rosa Astori, Eugenio Massa, Antonio Ponzetto

**Affiliations:** 1 Dipartimento Energia, Politecnico di Torino, Corso Duca degli Abruzzi 24, 10129 Torino, Italy; 2 Dipartimento di Oculistica, Azienda Ospedaliera Nazionale SS. Antonio e Biagio e Cesare Arrigo, Via Venezia 7, 15121 Alessandria, Italy; 3 Dipartimento di Scienze Mediche, Università di Torino, Corso A.M. Dogliotti 14, 10126 Torino, Italy

## Abstract

The aim of this work was to analyse the pressure inside the eyes anterior chamber, namedintraocular pressure (IOP), in relation to the biomechanical properties of corneas. The approach used was based on the constructal law, recently introduced in vision analysis. Results were expressed as the relation between the temperature of the ocular anterior chamber and the biomechanical properties of the cornea. The IOP, the elastic properties of the cornea, and the related refractive properties of the eye were demonstrated to be dependent on the temperature of the ocular anterior chamber. These results could lead to new perspectives for experimental analysis of the IOP in relation to the properties of the cornea.

In vision processing, images flow from the external environment to the brain. The information processed in this complex system during viewing was developed into a morphological network that can be modelled by the constructal law. Indeed, the brain architecture is closely linked to the information flow system, which is explained by stating that ‘the eyes scan the viewing area in the shortest time’^1^. Moreover, it has been emphasized that “The visual sensors and nerves in the retina are configured in the same way, as dendrites, in order to provide greater access between one surface (the retina) and one point (the optic nerve)”^1^.

This approach represents a new interesting method of analysing the complex systems that constitute vision and eyes because bio-engineering combines physical, chemical and applied mathematical results to study biological phenomena, and usually relates them to mechanical, electronic or information sciences. On the other hand, constructal law analyses the flows between the system and its environment, introducing a new viewpoint in engineering sciences^2^. Recently, by introducing this new basic and fundamental position into biochemical thermodynamics^3^,^4^,^5^,^6^, a biochemical engineering thermodynamic approach^7^,^8^ has been developed for complex biological systems, based on the analysis of interactions between a particular biological system and its environment. The result is a comprehensive understanding of the biophysical, biochemical and physiological processes in specific systems.

Here, we extend this approach to analysing the intraocular pressure (IOP), which is the fluid pressure inside the eye. Progressive loss-of-vision related to any IOP disorder is known as glaucoma, and it represents one of the major causes of blindness. In addition, IOP measurements are one of the most important medical parameters in postoperative evaluation for corneal, lenticular and vitreoretinal diseases.

There is a large amount of experimental evidence regarding the relevance of the central corneal thickness (CCT) in the measurement (tonometry) of the IOP^9^,^10^ and, therefore, measuring the CCT (pachymetry) has become standard in ophthalmological examinations in order to introduce a correction in the IOP evaluation. However, there are different algorithms for this correction which hold to different values in IOP corrections. But, a general method of analysis of the IOP is required in order to establish a unique correction of the IOP measurement. Indeed, biomechanical analyses of the physical properties of the eyes, in particular the cornea, are too complex to obtain a general mechanical model for linking the tissue morphology to their elastic properties and to the flows between the eye and its external environment^11^. The cornea tissue is characterized by the properties of the soft material, in particular^12^,^13^,^14^,^15^,^16^,^17^,^18^,^19^,^20^,^21^,^22^,^23^:

1. Heterogeneity in the central-to-peripheral, and anterior-to-posterior dimensions^12^;
2. Near incompressibility, due to the large amount of water and collagen fibres, with a consequent Poisson’s ratio of the cornea of about^13^ 0.5;
3. Non-linear constitutive behaviour in relation to the IOP variation^14^;
4. Viscoelastic and creep behaviour observed in cyclic tests^15^;
5. Structural anisotropy generated by the oriented collagen fibrils^16^.

Furthermore, biomechanical problems of the cornea are fundamental in certain diseases, such as^17^

1. Keratoconus, in relation to loss-of–rigidity in the cornea, and
2. Cornea ectasia after cornea refractive surgery, in relation to progressive cornea deformation due to impairment of the stroma.

In recent decades, measuring the cornea temperature has been shown to be indicative for many applications in diagnosing ocular disease^24^. On the other hand, there are plenty of models for the study of the cornea elastic properties and heat exchange, but there isn’t a general analytical models to link the cornea thermo-elastic properties with anterior chamber thermal behaviour. This correlation is in fact very important as the flow of aqueous humour in the anterior chamber is crucial for maintaining IOP, along with the shape and stability of the visual system, the diffusion of nutrients and the removal of metabolic waste^25^. The temperature gradient between the anterior and posterior surfaces of the anterior chamber causes buoyancy, which is the principal mechanism that causes anterior chamber flows^25^,^26^,^27^,^28^,^29^,^30^. In this context, we highlight that temperature is one of the principal regulators of tissue metabolism, and there is a continuous interest for analysing temperature in the study of ocular physiology and pathology^21^. However, to date there are no theoretical models able to support physicians in their analysis^21^.

Due to the theoretical difficulties in obtaining general results, a new approach can be adopted in order to analyse the properties of the cornea and the ocular anterior chamber in relation to IOP studies. To do so, we consider that flows of water (or in general, of liquids) constitute the IOP and, therefore, introducing the constructal law in the context of ophthalmological analysis of IOP is logical and interesting.

Consequently, in this investigation, we aimed to link constructal law to irreversible bioengineering thermodynamics in order to obtain a new approach for studying IOP, for analysing the relation between the cornea properties and the risk of glaucoma in hypertensive patients.

## Results

The constructal law is applied by analysing the effects of liquid flows in the ocular anterior chamber. Results are expressed as a relation between the temperature of the ocular anterior chamber and the environmental temperature.

**Figure 1:**
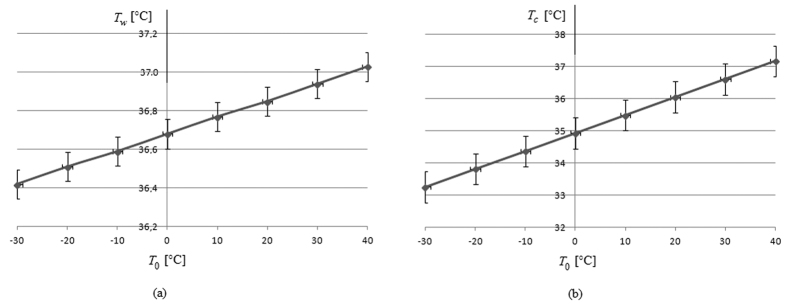
Temperature of the ocular anterior chamber and the cornea. Temperature of the ocular anterior chamber (**a**) and the cornea (**b**) were evaluated by using the equations (6 and 7) in relation to the variation of external environmental temperature.

**Figure 2:**
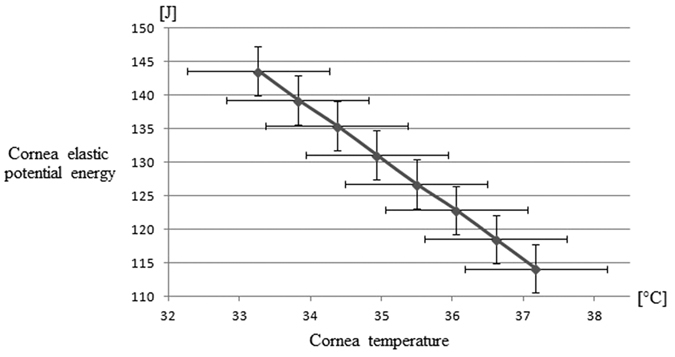
Elastic properties of the cornea as a function of cornea temperature.

Figure 1 represents the temperature of water inside the anterior chamber (a) and cornea temperature (b) in relation to the variation of the environmental temperature. We highlight that inside water temperature and cornea temperature growth with the growth of the environmental temperature. Figure 2 represents the cornea elastic potential energy in relation to cornea temperature. We highlight that the elastic potential energy decrease with the cornea temperature. Our results can be compared with the results obtained in refs 19,21. Our results agree with recent studies on humans^28^,^29^,^30^. Indeed, they highlighted that eyes with ischemic central venous retinal occlusion (determined by high pressure) have lower ocular surface temperature than non-ischemic eyes. It agrees with our result because the cornea potential energy is the work done by the border of the eye, and its magnitude is proportional to the magnitude of the intraocular pressure which causes the work against this surface. Consequently, lower the temperature greater the corneal potential energy and greater the intraocular pressure, with consequent ischemic consequences on the optic nerve.

They agree with the experimental results summarized in the literature^18^,^19^,^20^,^21^,^22^. It can be highlighted that the cornea temperature depends more on the environmental temperature than the anterior chamber.

Finally, we evaluated the elastic work (potential energy variation) of the cornea in relation to the cornea temperature. The work done by the cornea was found to decrease with the temperature. However, the cornea temperature is determined both by the environmental temperature and by the anterior chamber temperature. Therefore, there is a relation between the elastic work carried out by the cornea against the anterior chamber pressure and the anterior chamber temperature, consequently, there is a relation between the cornea thickness and the IOP. Our approach allows us to obtain this relation.

From equation (10), it can be highlighted that the difference between the inflow and outflow of liquids is the main cause of the variation in the elastic properties of the cornea and of the related IOP.

## Discussion

Glaucoma is a very serious optic neuropathy in which the destruction of ganglion cells and fibres determines an irreversible loss of visual field, and represents a cause of blindness. Controlling the IOP prevents patients from sustaining damage due to glaucoma and avoids blindness. Conversely, increasing IOP represents a primary risk factor for glaucoma development. Thermography has been used in the study of vascular factors in the physiopathology of glaucoma, revealing that ocular surface temperature can be used as an indicator of impaired retrobulbar haemodynamics in glaucoma studies^21^,^28^.

The cornea is the primary ocular refractive structure in the eye composed of five layers (epithelium, Bowman’s layer, stroma, Descemet’s membrane, and endothelium). It has a radius of 10–11 mm and a thickness of 0.5 mm^17^.

The cornea exchanges heat with the environment and with the anterior chamber; consequently, its surface temperature results as being underlying in maintaining the temperature of the anterior chamber constant. The temperature of the cornea can be obtained by considering the conductive heat transfer across the cornea and, in this way, a relation between the cornea temperature and the anterior chamber is obtained. Moreover, its elastic properties affect the value of the IOP; indeed, in 1957, Goldmann and Schmidt introduced^23^ the hypothesis that the IOP is underestimated in patients with thin corneas, while it is overestimated in patients with thick corneas. A correlation between the cornea thickness and the IOP has been experimentally verified^29^, and can be attributed to the fact that the IOP is the sum of the effects of:

1. The swelling pressure (SP), the repulsion caused by the negatively charged stromal glycosaminoglycans, and
2. The imbibition pressure (IP), the negative pressure that causes the fluid inflow into the cornea (IOP = SP + IP).

From our results, we can state that:

1. There is a relation between the temperature of the ocular anterior chamber and the IOP;
2. There is a relation between the cornea temperature and the temperature of the ocular anterior chamber;
3. There is a relation between the cornea temperature and its elastic behaviour;
4. There is a relation between the IOP and the cornea thickness, which can be evaluated by measuring the ocular anterior temperature.

The first principle of thermodynamics can be introduced to evaluate the work done by the anterior chamber against the internal cornea wall. The temperature of the aqueous humour is related to the difference between the heat dissipated across the cornea and the heat generated by metabolic activities and the vascular supply to the anterior chamber^22^. Thus, temperature plays a role in regulating the aqueous humour secretion, excretion, and flow dynamics, with consequences on the IOP regulation and the relative diseases, such as glaucoma. Moreover, the correlation between the anterior chamber temperature and the cornea temperature can be utilised for analysing the tear film evaporation and its relation to the environmental conditions. Therefore, it can be assumed that the transient receptor potential channel isotopes may play a crucial role in the control of aqueous humour dynamics, as suggested by Fabiani^21^. As such, temperature can be introduced as a new additional target in the control of glaucoma progression in ocular hypertensive patients. It should be highlighted that there is a trend for ocular temperature to decrease with age^24^ and it is therefore important to introduce these new approaches by evaluating individual patients with relation to his/her personal evolution. Finally, a relevant relationship between cornea elastic properties, refractive errors, and anterior chamber properties has been experimentally highlighted^29^, confirming our theoretical results: a recent study on humans highlighted that eyes with ischemic central venous retinal occlusion (CRVO) have lower ocular surface temperature than non-ischemic eyes^30^. Last, Galassi *et al*. has defined ocular surface temperature as a marker of impaired retrobulbar hemodynamics in patient with glaucoma^28^.

So, from the constructal law, we can introduce the analysis of liquid fluxes in the ocular anterior chamber. Variation of the mass flows in the anterior chamber cause an alteration in IOP and, consequently, in the elastic properties of the cornea. However, the cornea properties affect the refractive properties of the eye and, as a result, a variation in IOP could also be assessed by evaluating the vision itself. Indeed, IOP depends only on the flows of aqueous humour which is a balance between secretion and excretion. Three mechanisms are involved in the acqueous humour formation: diffusion, ultrafiltration, and active secretion. Constructal law is a physical approach based on the analysis of the flows. It is a new approach introduced in thermodynamics by Adrian Bejan in order to explain optimal shapes of natural structures^31^. This law is finding its applications from engineering to economics, from physics to biology, from hydraulics to geology, from urban designing to physiology, etc.^32^,^33^,^34^,^35^,^36^,^37^. The constructal law describes how Nature and engineers “design” a system, so it is very useful to understand the bases of the biosystems and their interaction with the environment through flows.

In conclusion, there exists a correlation between IOP and temperature. Temperature can be measured by non-contact measurements systems. This characteristic is interesting to design non contact tonometers for future home patients controls and related personalized therapies.

## Methods

In order to extend a constructal approach from the analysis of vision to the analysis of the eyes, we considered that the flows related to IOP are water inflows and outflows between the ocular anterior chamber and blood vessels. Therefore, considering the eye as an open system in relation to the body, we apply the first law of thermodynamics^38^:


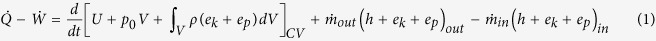


where 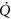 is the heat power exchanged between the cornea and the external environment, 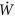 is the mechanical power carried out by the tissue in relation to the pressure and elastic properties of the cornea, *U* is the internal energy, *p*_0_ is the external environmental pressure, *V* is the volume of the anterior chamber, *ρ* is the water density, *e* is the specific energy, *k* stands for kinetic, *p* as a subscript means potential, *h* is the specific enthalpy of the water, *in* means inflow, *out* means outflow, and *CV* means control volume. The kinetic energy and the potential energy are so small that they can be considered null, and we analysed the behaviour of the eye during the time of observation, so that we integrated time, obtaining:


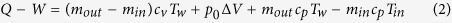


where *c*_*v*_ is the specific heat at a constant volume of the water inside the anterior chamber, *c*_*p*_ is the specific heat at a constant pressure of the water, *T*_*w*_ is the temperature inside the anterior chamber, *T*_*in*_ = 37 °C is the temperature of the inflow water, *m*_*w*_ is the mass, *T*_0_ is the external environmental temperature, considered in the range [−30 °C, 40 °C], and Δ*V* is the volume variation due to water stagnation in the ocular anterior chamber. Then, considering that the anterior chamber exchanges heat with the environment by the cornea surface, we can write:


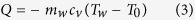


obtaining:


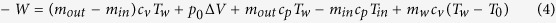


Then, we can evaluate the work carried out by the water against the external surface of the anterior chamber as^38^:


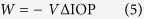


and the ocular anterior chamber temperature results can be obtained:


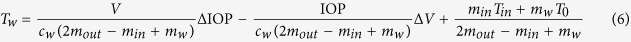


where *c*_*w*_ = *c*_*p*_ = *c*_*v*_ is the specific heat of the liquid water. In an eye without any pressure diseases *m*_*out*_ ≈ *m*_*in*_, *p*_0_ ≈ IOP as it represents the pressure of the interior chamber against the elasticity of the cornea back wall.

Starting from this result, we can consider that the anterior chamber exchanges heat through the cornea surface. The heat transfer mechanisms are the conduction across the cornea length, and the convection with the environmental air on the cornea external surface. So, the value of the cornea temperature *T*_*c*_ can be obtained by the usual heat transfer approach^18^, as follows:


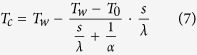


where^18^,^19^,^20^,^21^ *s* is the mean cornea thickness (0.5 mm), *λ* is the cornea conductivity (0.6 W m^−1^ K^−1^) and *α* is the convective heat transfer coefficient (50 W m^−2^ K^−1^). The results obtained are represented in Fig. 1. It should be noted that the cornea temperature is more affected by the environmental temperature and that the cornea temperature is crucial in relation to the variation of the anterior chamber temperature. The data obtained agrees with the data in the literature^17^,^18^,^19^,^20^,^21^,^22^,^30^, as discussed in the previous section, with an error of less than 1% (see refs 19,21). The time of observation considered is greater than the thermal transient, evaluated^19^ in 5000 s.

Now considering the extended Bernoulli’s equation^38^:


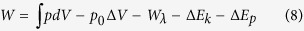


where *W*_*λ*_ is the work lost for irreversibility, *E*_*k*_ is the kinetic energy and *E*_*p*_ is the cornea elastic potential energy, and introducing this into the relation (4) it follows that the elastic potential variation of the cornea results as:


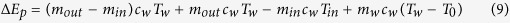


This result is represented in Fig. 2. The shape has been obtained considering the kinetic energy and the work lost for irreversibility as being null. It should be emphasized that the cornea elastic work (variation of the elastic potential energy) depends on the temperature of the cornea.

Finally, considering the relation (10) and the relation (6) it follows that


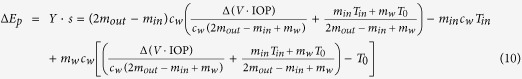


where *Y* is the Young modulus and *s* is the cornea thickness.

## Additional Information

**How to cite this article**: Lucia, U. *et al*. Constructal approach to bio-engineering: the ocular anterior chamber temperature. *Sci. Rep.* **6**, 31099; doi: 10.1038/srep31099 (2016).

## References

[b1] Bejan A. The Golden Ratio predicted: vision, cognition and locomotion as a single design in Nature. Int. J. Design & Nature and Ecodynamics 4, 97–104 (2009).

[b2] Bejan A. The Physics of Life: The Evolution of Everything (St. Martin’s Press, New York, 2016).

[b3] Hatsopoulos G. N. & Keenan J. H. Principles of General Thermodynamics (Wiley, New York, 1965).

[b4] Kestin J. A Course in Thermodynamics (McGraw-Hill, New York, 1979).

[b5] Alberty R. Thermodynamics of Biochemical Reactions (Wiley, New York, 2003).

[b6] Avery J. S. Information, Theory and Evolution (World Scientific, London, 2012).

[b7] Lucia U. Bioengineering thermodynamics: an engineering science for thermodynamics of biosystems. IJoT 18, 254–265 (2015).

[b8] Lucia U. Bioengineering thermodynamics of biological cells. Theor. Biol. Med. Model 12, 29 (2015). 26620568 10.1186/s12976-015-0024-z PMC4666120

[b9] Ehlers N., Hansen F. K. & Aasved H. Biometric correlations of corneal thickness. Acta Ophthalmol. 53, 652–659 (1975). 1242286 10.1111/j.1755-3768.1975.tb01784.x

[b10] Johnson M., Kass M. A., Moses R. A. & Grodzki W. J. Increased corneal thickness simulating elevated intraocular pressure. Arch. Ophthalmol. 96, 664–665 (1978). 646694 10.1001/archopht.1978.03910050360012

[b11] Fung Y. C. Biomechanics: Mechanical properties of living tissues 2^nd^ Ed. (Springer-Verlag, New York, 1993).

[b12] Dupps W. J. & Wilson S. E. Biomechanical and wound healing in the cornea. Exp. Eye Res. 83, 709–720 (2006). 16720023 10.1016/j.exer.2006.03.015 PMC2691611

[b13] Uchio E., Ohno S. Kudoh J., Aoki K. & Kisielewicz L. T. Simulation model of an eyeball based on finite element analysis on a supercomputer. Br. J. Ophthalmol. 83, 1106–1111 (1999). 10502567 10.1136/bjo.83.10.1106 PMC1722840

[b14] Nyquist G. Rheology of the cornea: Experimental techniques and results. Exp. Eye Res. 7, 183–188 (1968). 5646606 10.1016/s0014-4835(68)80064-8

[b15] Hoeltzel D., Altman P., Buzard K. & Choe K. Strip extensiometry for comparison of the mechanical response of bovine, rabbit, and human corneas. J. Biomech. Eng. 114, 202–215 (1992). 1602763 10.1115/1.2891373

[b16] Meek K. M. & Boote C. The organization of collagen term in the corneal stroma. Curr. Eye Res. 78, 503–512 (2004). 10.1016/j.exer.2003.07.003 15106929

[b17] Bolivar G., Moreno-Arrones J. P. & Teus M. A. Cornea and Glaucoma. In Rumelt S. Ed. Glaucoma – Basic and clinical aspects (InTech, Rijeka, 2013).

[b18] Braakman S. T., Moore J. E., Ethier C. R. & Overby D. R. Transport across Schlemm’s canal endothelium and the blood-aqueous barrier. Exp. Eye Res. 146, 17–21 (2016) 26689753 10.1016/j.exer.2015.11.026 PMC4893895

[b19] Shafahi M. & Vafai K. Human eye response to thermal disturbances. J. Heat. Transf. 133, 011009 (2011).

[b20] Gokul K. C., Gurung D. B. & Adhikary P. R. FEM Approach for Transient Heat Transfer in Human Eye. Appl. Math. 4, 30–36 (2013).

[b21] Fabiani C., Li Voti R., Rusciano D., Mutolo M. G. & Pescosolido N. Relationship between corneal temperature and intraocular pressure in healthy individuals: A clinical thermographic analysis. J. Ophthal. 2016, 3076031 (2016). 26904273 10.1155/2016/3076031 PMC4745937

[b22] Mapstone R. Determinants of corneal temperature. Br. J. Ophthal. 52, 729–741 (1968). 5686964 10.1136/bjo.52.10.729 PMC506681

[b23] Goldmann H. & Schmidt T. Uber applanationstonometrie. Ophthalmologie 134, 221–242 (1957). 10.1159/000303213 13484216

[b24] Morgan P. B., Soh M. P. & Efron N. Corneal surface temperature decreases with age. Cont. Lens Anterior Eye 22, 11–13 (1999). 16303398 10.1016/s1367-0484(99)80025-3

[b25] Avtar R. & Srivastava S. The convection flow of aqueous humor in the anterior chamber of human eye. Adv. Appl. Sci. Res. 5, 359–369 (2014).

[b26] Ehrlich P. Ueber provocirte Fluorescenzerscheinungen am Auge. Deutsch. med. Wschr. 8, 35–37 (1882).

[b27] Wyatt H. J. Ocular Pharmacokinetics and Convectional Flow: Evidence from Spatio-Temporal Analysis of Mydriasis. Ocul. Pharmacol. Therapeut. 12, 441–459 (1996). 10.1089/jop.1996.12.441 8951681

[b28] Galassi F., Giambene B., Corvi A. & Falaschi G. Evaluation of ocular surface temperature and retrobulbar haemodynamics by infrared thermography and ocular Doppler imaging in patients with glaucoma. Br. J. Ophtal. 91, 878–881 (2007). 10.1136/bjo.2007.114397 PMC1955655 17314146

[b29] Chen M.-J., Liu Y.-T., Tsai C.-C., Chen Y.-C., Chou C.-K. & Lee S.-M. Relationship between central cornea thickness, refractive error, corneal curvature, anterior chamber depth and axial length. J. Chin. Med. Ass. 3, 133–137 (2009). 19299220 10.1016/S1726-4901(09)70038-3

[b30] Sodi A., Giambene B., Falaschi G., Caputo R., Innocenti B., Corvi A. & Menchini U. Ocular surface temperature in central retinal vein occlusion: preliminary data. Eur. J. Ophtalmol. 17, 755–759 (2007). 10.1177/112067210701700511 17932851

[b31] Bejan A. Shape and Structure, from Engineering to Nature (Cambridge University Press, Cambridge, UK, 2000).

[b32] Bejan A. Rolling stones and turbulent eddies: why the bigger live longer and travel farther. Sci. Rep. 6, 21445 (2016). 26883787 10.1038/srep21445 PMC4756689

[b33] Bejan A. Why humans build fires shaped the same way. Sci. Rep. 5, 11270 (2015). 26053318 10.1038/srep11270 PMC4459218

[b34] Bejan A. Why the bigger live longer and travel farther: animals, vehicles, rivers and the winds. Sci. Rep. 2, 594 (2012). 22924107 10.1038/srep00594 PMC3426796

[b35] Bejan A., Lorente S., Yilbas B. S. & Sahin A. Z. Why solidification has an S-shaped history. Sci. Rep. 3, 1711 (2013).

[b36] Bejan A. Maxwell’s Demons Everywhere: Evolving Design as the Arrow of Time. Sci. Rep. 4, 4017 (2014). 24510201 10.1038/srep04017 PMC3918919

[b37] Bejan A., Ziaei S. & Lorente S. Evolution: Why all plumes and jets evolve to round cross sections. Sci. Rep. 4, 4730 (2014). 24756029 10.1038/srep04730 PMC3996479

[b38] Borgnakke C. & Sonntag R. E. Fundamentals of thermodynamics (John Wiley & Sons, Hoboken, 2009).

